# Successful treatment of anti-EPO antibody associated refractory anemia with hypoxia-inducible factor prolyl hydroxylase inhibitor

**DOI:** 10.1080/0886022X.2020.1803087

**Published:** 2020-08-19

**Authors:** Huifang Zhang, Zineng Huang, Liyu He, Fang Yuan, Lin Sun, Fuyou Liu, Li Xiao

**Affiliations:** Department of Nephrology, The Second Xiangya Hospital, Central South University, Changsha, Hunan, China

Dear Editor,

Renal anemia is one of the most commonly documented complications of chronic kidney disease (CKD) patients with hemodialysis and occurs as kidneys lose the ability to generate adequate endogenic erythropoietin (EPO). The most frequent treatments for renal anemia are administration of Recombinant human Erythropoietin (rHuEPO) and iron supplementation [1]. However, an increasing incidence of refractory anemia is found among CKD population and many factors contribute to this refractoriness, such as oxidative stress, older age, EPO antibody, neutrophil and monocyte activation, decreased iron absorption and utilization, vitamin deficiencies, hyperparathyroidism and aluminum toxicity [2–6]. Effective treatments for refractory renal anemia are seldom reported. Notably, roxadustat, a small molecule inhibitor of hypoxia-inducible factor prolyl hydroxylase (HIF-PHI) newly approved in China, has been demonstrated to be applicable for the treatment of renal anemia in patients with or without dialysis. However, its efficacy in renal anemia patients with EPO resistance remains to be unveiled. Here, we present a case of beneficial effects of roxadustat against refractory renal anemia associated with anti-EPO antibody and significantly elevated ferritin, which might offer a new perspective on dealing with refractory renal anemia.

A 56-year-old woman (weight, 82 kg; BMI 30.86) was first admitted to our hospital in May, 2016 because of elevation of blood glucose for 22 years with a high level of serum creatinine for 5 years, fatigue for 5 months and dizziness for 20 days. In the past 3 years, the patient had been receiving regular dialysis (biw or tiw), iron supplement and rHuEPO in another hospital. There was no family history of renal diseases. Physical examinations showed slightly pale conjunctiva and nail bed, moderate lower limbs edema and high blood pressure at 170/95 mmHg. Laboratory evaluations revealed a moderate anemia with Hb 72 g/L and reticulocyte count 3.53% (reference range, 0.80–2.10%), iron overload with ferritin >2000 μg/L, no deficiency of folate, increasing percentage of neutrophils with normal white blood cell 3.8 ×$10^{9}$/L (reference range, 3.50–9.50 ×$10^{9}$/L), thrombocytopenia with platelet 100 ×$10^{9}$/L; and hypoproteinemia (Table 1). Other laboratory data were as follows: glycosylated hemoglobin A1c 8.50% (reference range, 3.9–6.10%), serum creatinine 975.8 μmol/L (reference range, 44–133 μmol/L) and blood urea nitrogen 20.12 mmol/L (reference range, 2.90–7.14 mmol/L), intact parathyroid hormone (0 min) 15.47 pmol/L (1.60–6.90 pmol/L), calcium 2.07 mmol/L (2.03–2.54 mmol/L), phosphorus 1.98 mmol/L (0.90–1.34 mmol/L). Coombs test, antinuclear antibody, extractable nuclear antigen, antineutrophil cytoplasmic antibodies, monoclonal gammopathy, tumor markers C12, hepatitis B virus and hepatitis C virus were negative. Complement C3 and C4 levels were normal. Abdominal B-mode ultrasound indicated that bilateral kidneys was relatively small and echo increased and that there was no hypersplenism. Therefore, the patient was diagnosed with diabetic nephropathy, renal anemia and hypertension. Regular hemodialysis (biw or tiw) with a Kt/V of 1.5 and low molecular weight heparin for anticoagulation, rHuEPO (4000 IU SC tiw) and antihypertensive drug (levamlodipine 5 mg po qd, extended-release metoprolol succinate 47.5 mg po qd, olmesartan 20 mg po qd) were given. Considering the elevation of ferritin level, iron supplement was not introduced.

**Table 1.** Partial biochemical indicators from May 2016 to June 2020.

Time	Complete blood count							Iron metabolism				Inflammation		Other biochemical indexes		
	RBC^a^	Hb^b^	MCV^c^	MCH^d^	MCHC^e^	N%^f^	PLT^g^	SF^h^	Fe^i^	TASR^j^	TIBC^k^	PCT^l^	CRP^m^	Fol ^n^	Vit B12^o^	ALB^p^
2016-05	2.08	72	100.5	34.6	344	82.4	100	>2000	–	–	–	19.35	–	>20	–	38.6
2018-04	1.52	52	101.3	34.2	388	81.9	65	3706	12.9	44.6	28.9	0.580	2.33	>20	>2000	38.2
2019-06	1.76	50	96.6	32.2	333	91.1	60	3800	–	–	–	1.920	3.05	>20	1231	41.3
2019-10	2.80	97	105.2	32.2	306	87.8	94	1004	19.8	46.5	42.6	–	4.13	>20	1304	38.8
2020-06	3.86	133	99.2	34.5	347	81.4	126	877	–	–	–	0.557	3.77	>20	809	38.3

^a^RBC, red blood cell, reference range, 3.80–5.10 × $10^{12}$/L; ^b^Hb, hemoglobin, reference range, 115–150 g/L; ^c^MCV, mean corpuscular volume, reference range, 82–100 fL; ^d^MCH, mean corpuscular hemoglobin, reference range, 27–34 pg; ^e^MCHC, mean corpuscular hemoglobin concentration, reference range, 316–354 g/L; ^f^N%, percentage of neutrophils, reference range, 40–75%; ^g^PLT, platelet count, reference range, 125–350 ×${\;10}^{9}$/L; ^h^SF, serum ferritin, reference range, 4.3–29 μg/L; ^i^Fe, serum iron, reference range, 7.8–32.2 μmol/L; ^j^TASR, transferrin saturation, reference range, 20.0–55.0 μmol/L; ^k^TIBC, total iron binding capacity, reference range, 54.0–77.0 μmol/L; ^l^PCT, procalcitonin, reference range, <0.1 ng/mL; ^m^CRP, C-reactive protein, reference range, 0.00–8.00 mg/L; ^n^Fol, folate, reference range, 3.10–20.50 ng/mL; ^o^VitB12, vitamin B12, reference range, 187.0–883.0 pg/mL; ^p^ALB, serum albumin, reference range, 40.0–55.0g/L.

However, in April 2018, hematologic parameters of anemia were not improved and even gradually worsened, with Hb level of 52 g/L (Table 1, Figure 1) in the case of no active bleeding. Laboratory values showed deteriorating iron metabolism with markedly elevated ferritin 3706 μg/L, decreased total iron binding capacity, normal serum iron and transferrin saturation, and no severe chronic inflammation assessed by C-reactive protein (CRP) and procalcitonin (PCT). (Table 1) We evaluated inflammatory markers again 6 months later and confirmed mild inflammation: percentage of neutrophils, PCT, and CRP were 85.00%, 1.010 ng/mL, and 6.08 mg/L, respectively. We further conducted a series of hematological examinations to exclude autoimmune hemocytopenia, myelodysplastic syndromes and cancers. Laboratory examination results were as follows: antigens associated with autoimmune hemocytopenia were negative; fluorescence *in situ* hybridization of bone marrow samples was negative (Figure 2); chromosomal karyotype analysis of bone marrow samples revealed normal karyotypes; screening for 34 high frequency gene mutations in myeloid blood diseases showed that splice site c.2408 + 1G > A in gene DNMT3A mutated, which is associated with acute myelocytic leukemia, myelodysplastic syndromes, myeloproliferative neoplasms and chronic myelomonocytic leukemia; in December, 2018, the first finding on bone marrow cytology was active proliferation and erythroid cells increased, resulting a decrease percentage of lymphocyte; distribution of megakaryocytes and platelets were normal (Figure 3(a) and Figure 3(b)); pathological examinations of bone marrow samples found that there were no abnormal pathological cells (Figure 3(c)); iron stain of bone marrow samples showed extracellular iron +, intracellular iron 55%, no ring sideroblasts (Figure 3(d)); the patient did not receive any special treatment, but the second finding on bone marrow cytology returned to normal in March, 2019, with granulocyte 59%, erythroid 27%, granulocyte: erythroid = 2.2:1. Notably, we discovered elevated EPO level 53.90 (reference range, 4.3–29 μg/L) and positive EPO antibody in May, 2018, indicating the presence of EPO resistance; two companies detected EPO level and EPO antibody respectively through multiple blood samplings that were collected at the same time. She was started on methylprednisolone (24 mg po qd) because we suspected that this patient had autoimmune hemocytopenia; however, negative antigens associated with autoimmune hemocytopenia excluded the diagnosis after 1 month, so methylprednisolone was stopped. Therefore, we transfused erythrocytes, elevated rHuEPO dose (4000 IU SC tiw to 6000 IU SC tiw) and started deferasirox (1125 mg po qd) to the patient.

**Figure 1.**
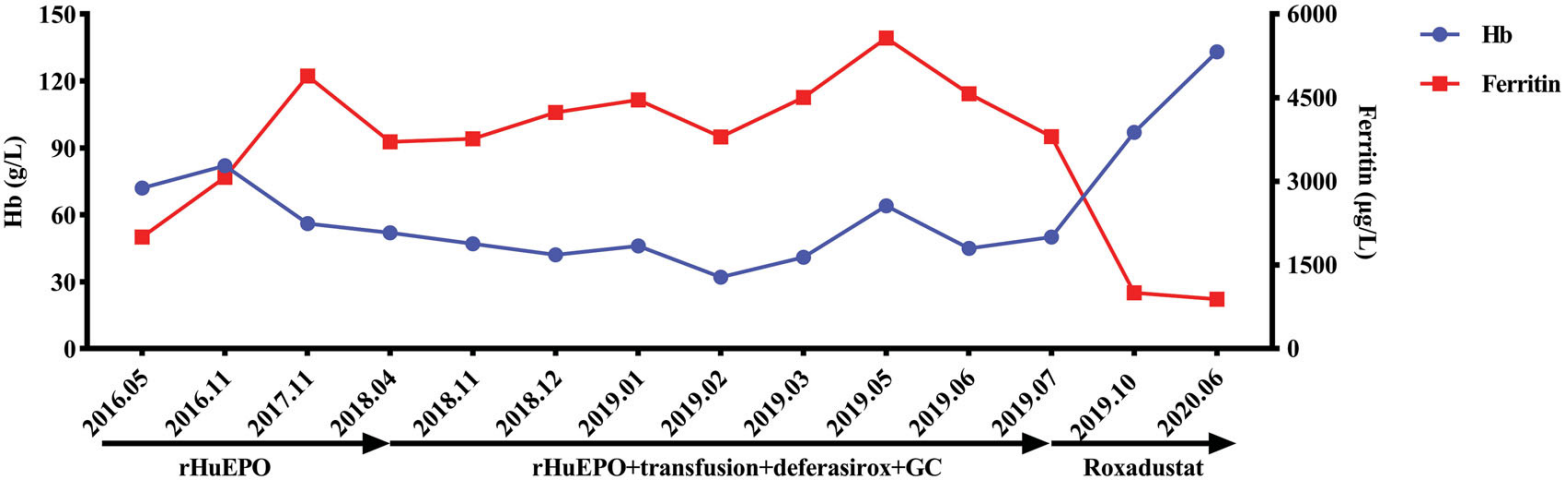
Evolution of hemoglobin (Hb) and ferritin with anti-anemia treatment from May, 2016 to June, 2020. From May, 2016 to April, 2018, the patient had been intermittently receiving rHuEPO for renal anemia. From April, 2018 to June, 2019, the patient was treated with rHuEPO, transfusion, deferasirox and glucocorticoid for refractory anemia, iron overload, positive autoantibodies of red blood cells and EPO-antibody. However, Hb had been less than 90 g/L, even lower than 60 g/L, and ferritin had been above 2000 μg/L. In July, 2019, the patient was prescribed roxadustat for EPO-resistance renal anemia. Three months later, Hb increased to 97 g/L, and ferritin decreased to 1004 μg/L. After consumption of roxadustat for one year, Hb was 133 g/L, and ferritin was 887 μg/L. (GraphPad Prism was applied to create the picture.)

**Figure 2.**
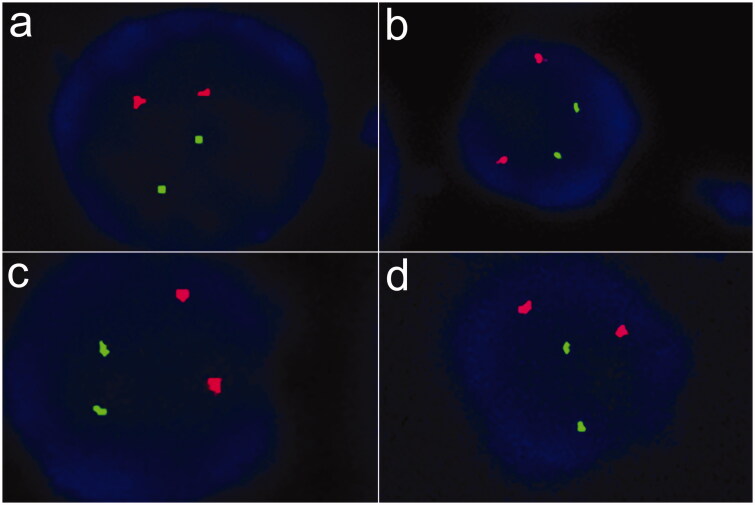
Fluorescence *in situ* hybridization of bone marrow samples for myelodysplastic syndrome. (a) 99% cells showed two green and two red signals with GLP D5S23, D5S72\EGR1 probe, indicating that the patient was without absence of 5q31. (b) 99% cells showed two green and two red signals with GLP D5S23, D5S72\CSF1R probe, indicating that the patient was without absence of 5q33. (c) 98% cells showed two green and two red signals with GLP D7S486, D7S522/CSP7 probe, indicating that the patient was without absence of chromosome 7. (d) 99% cells showed two green and two red signals with GLP D20S108/CSP8 probe, indicating that the patient was without absence of chromosome 8 and 20q12.

**Figure 3.**
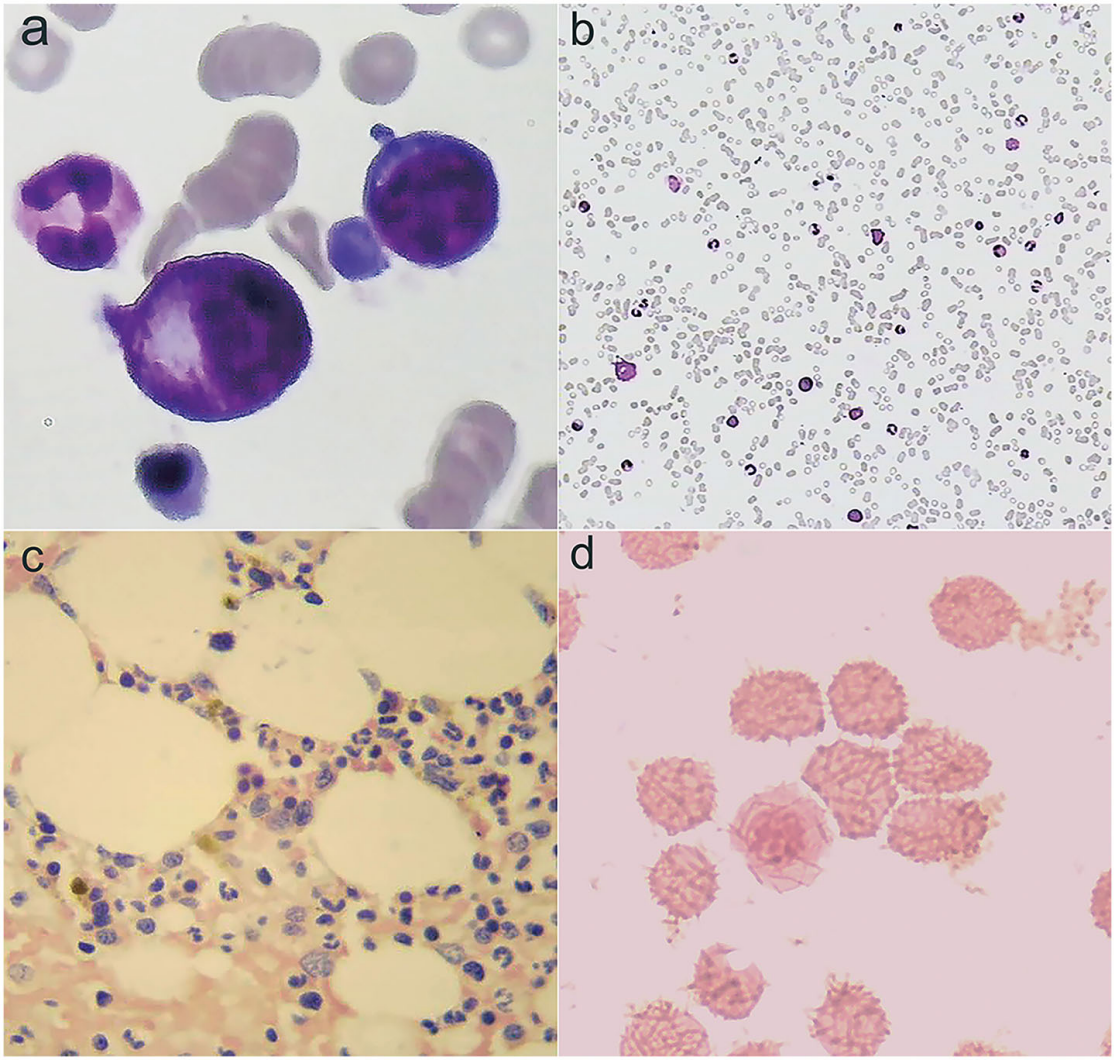
Bone marrow smears. (a,b) Cytologic examination in December, 2018: bone marrow was active proliferation, granulocyte was 49%, erythroid was 45%, and granulocyte: erythroid was 1.1:1. (c) Pathological examination: no pathological cells. (d) Iron stain: extracellular iron +, intracellular iron 55%, no ring sideroblasts.

In June, 2019, we discovered that anemia, obstacle to iron utilization and thrombocytopenia of the patient continued to deteriorate, with Hb 50 g/L, ferritin 3800 ng/mL, and PLT 60 ×${\;10}^{9}$/L (Table1, Figure 1). Therefore, in July, 2019, due to poor response to the conventional treatment, we prescribed roxadustat (100 mg po tiw) for the patient with refractory renal anemia; and deferasirox was stopped. Fortunately, dizziness and fatigue achieved remarkable remission after continuous administration of roxadustat for three months. Physical examinations found that conjunctiva and nail bed were red. In addition, laboratory indexes of anemia and thrombocytopenia significantly improved, with elevated levels of Hb 97 g/L and PLT 94 × $10^{9}$/L, although inflammation state still existed, with a high level of percentage of neutrophils 87.80%. Encouragingly, the level of ferritin markedly declined to 1004 μg/L. After one year of roxadustat treatment, the patient was with Hb 133 g/L, red blood cells 3.86×$10^{12}$/L, ferritin 887 ng/mL, PLT 127 ×${\;10}^{9}$/L and percentage of neutrophils 81.4% (Table1), indicating that roxadustat may play a remarkable role in this patient regarding improving anemia and regulating iron metabolism. In addition, the patient showed no any adverse events.

Renal anemia is among the most common complications of CKD with erythropoiesis stimulating agents as basic therapeutic drugs. However, EPO resistance frequently occurs in CKD patients. HIF-PHI represents a novel pharmacological treatment of renal anemia by stabilizing hypoxia-inducible factor [7,8]. Several clinical trials have demonstrated the safety and efficiency of HIF-PHI in kidney disease patients with or without long-term dialysis [9,10]. Efficiency of HIF-PHI against refractory renal anemia associated with EPO resistance and disordered iron metabolism has not been reported yet. The patient was very likely to develop anti-EPO antibody and an extremely high ferritin level due to EPO therapy and iron supplement for 3 years in another hospital. EPO resistance in the patient may be due to anti-EPO antibody, obstacle to iron utilization and chronic inflammation. Apparent improvement of Hb and iron metabolism of this patient indicates that HIF-PHI may works effectively in renal anemic patients with EPO resistance via its dual anti-anemic mechanisms: stimulating endogenous erythropoietin synthesis – may not be neutralized by exogenous rHuEPO inducing anti-EPO antibody, and downregulating hepcidin. Platelet count improved significantly after roxadustat treatment while currently no literatures show that platelet production is associated with erythroid or roxadustat. Therefore, the reason for platelet reduction and improvement in this patient need to be further investigated.

In summary, the case shows that refractory renal anemia developing in response to anti-EPO antibodies and disordered iron metabolism can be treated with roxadustat.

## Data Availability

All data supporting the case are included in the manuscript.
